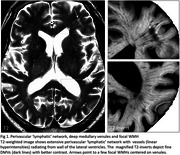# Cerebral focal WMH co‐locate with transcerebral intramedullary vessels and can vary over time

**DOI:** 10.1002/alz70856_099743

**Published:** 2025-12-25

**Authors:** Fuqiang Gao, Joel Ramirez, Melissa F Holmes, Julia Keith, Mario Masellis, Richard H. Swartz, Sandra E. Black

**Affiliations:** ^1^ Dr. Sandra Black Centre for Brain Resilience and Recovery, Sunnybrook Research Institute, Toronto, ON, Canada; ^2^ Dr. Sandra E. Black Centre for Brain Resilience and Recovery, LC Campbell Cognitive Neurology, Hurvitz Brain Sciences Program, Sunnybrook Research Institute, University of Toronto, Toronto, ON, Canada; ^3^ University of Toronto Scarborough, Toronto, ON, Canada; ^4^ Sunnybrook Health Sciences Centre, Toronto, ON, Canada; ^5^ Division of Neurology, Department of Medicine, Sunnybrook Health Sciences Centre, Toronto, ON, Canada; ^6^ Cognitive and Movement Disorders Clinic, Sunnybrook Health Sciences Center, Toronto, ON, Canada; ^7^ Sunnybrook Research Institute, Toronto, ON, Canada; ^8^ University of Toronto, Toronto, ON, Canada; ^9^ Hurvitz Brain Sciences Program, Toronto, ON, Canada; ^10^ Sunnybrook Health Sciences Centre, University of Toronto, Toronto, ON, Canada

## Abstract

**Background:**

The origin of MRI focal white matter hyperintensities (fWMH) is not fully understood. Recent evidence suggests the perivascular surface of arterioles and venules may serve as cerebral lymphatics for homeostasis of interstitial fluid and toxic metabolite clearance. This system could be injured by age‐related vascular wall damage, particularly venous collagenosis in Alzheimer's disease (AD), where fWMH could be an early marker of this injury. We investigated whether fWMH spatially co‐localize with deep medullary vessels (DMV), and change dynamically reflecting vasogenic edema.

**Method:**

107 AD and 30 controls (age=74) were included with baseline and follow‐up MRI. fWMH were foci <10mm on T2/FLAIR. DMVs were linear visible streaks on T1, invert‐T2 or SWI. T2/FLAIR was co‐registered to T1 space. The spatial relationship of each fWMH with DMV was classified as either 'perivascular positive' if an fWMH was overlapped/centered by a DMV or otherwise as 'perivascular negative'. Each fWMH was followed for change over time in 70 AD and 19 controls. We reasoned that fWMH would exhibit dynamic change if they reflect vasogenic edema.

**Result:**

At the baseline, 1630 fWMH were identified, with 91.6% perivascular positive. They distributed mostly in the frontal (60.2%) and occipitoparietal (32.2%) region, along the angles of the lateral ventricles, areas with highest distribution of intramedullary venules, suggesting perivenular distribution. In follow‐up, 1098 fWMH showed change over time. 1019 (92.8%) of 1098 fWMH were perivascular positive with 8.5% decreased, 31.9% increased and 59.6% unchanged over 1.5 years. AD had a higher rate of fWMH increase (χ^2^=6.23, *p* = 0.012), while controls had a higher rate of fWMH unchanged over time (χ^2^=5.16, *p* = 0.023). Most DMVs connected to lateral ventricles and had trans‐cerebral features, consistent with intramedullary veins. Small venular infarctions were sometimes observed.

**Conclusion:**

Most fWMH were distributed along DMVs, particularly. Their dynamic progression was compatible with being fluid in nature and not necessarily indicative of ischemiaas previously thought. fWMH could relate to multiple underlying pathologies, but venous insufficiency of deep intramedullary venules may be an important substrate of fWMH in AD and aging.